# A Pediatrics Utilization Study in The Netherlands to Identify Active Pharmaceutical Ingredients Suitable for Inkjet Printing on Orodispersible Films

**DOI:** 10.3390/pharmaceutics12020164

**Published:** 2020-02-17

**Authors:** J. Carolina Visser, Lisa Wibier, Olga Kiefer, Mine Orlu, Jörg Breitkreutz, Herman J. Woerdenbag, Katja Taxis

**Affiliations:** 1 Department of Pharmaceutical Technology and Biopharmacy, University of Groningen, Antonius Deusinglaan 1, 9713 AV Groningen, The Netherlands; l.wibier@student.rug.nl (L.W.); h.j.woerdenbag@rug.nl (H.J.W.); 2 Department of PharmacoTherapy, Epidemiology and Economics, University of Groningen, Antonius Deusinglaan 1, 9713 AV Groningen, The Netherlands; k.taxis@rug.nl; 3 Institute of Pharmaceutics and Biopharmaceutics, Heinrich-Heine-University Düsseldorf, 40225 Düsseldorf, Germany; olga.kiefer@hhu.de (O.K.); Joerg.Breitkreutz@hhu.de (J.B.); 4 School of Pharmacy, University College London, London WC1N 1AX, UK; m.orlu@ucl.ac.uk

**Keywords:** drug utilization research, pediatrics, orodispersible films, pharmaceutical inkjet printing, pharmacy

## Abstract

Background: The use of medication in pediatrics, children aged 0–5 years, was explored so as to identify active pharmaceutical ingredients (APIs) suitable for inkjet printing on a plain orodispersible film (ODF) formulation in a pharmacy. Methods: The database IADB.nl, containing pharmacy dispensing data from community pharmacies in the Netherlands, was used to explore medication use in the age group of 0–5 years old, based on the Anatomical Therapeutic Chemical classification code (ATC code). Subsequently, a stepwise approach with four exclusion steps was used to identify the drug candidates for ODF formulation development. Results: there were 612 Active Pharmaceutical Ingredients (APIs) that were dispensed to the target group, mostly antibiotics. Of the APIs, 221 were not registered for pediatrics, but were used off-label. After the exclusion steps, 34 APIs were examined regarding their suitability for inkjet printing. Almost all of the APIs were sparingly water soluble to practically insoluble. Conclusion: Pharmaceutical inkjet printing is a suitable new technique for ODF manufacturing for pediatric application, however the maximal printed dose as found in the literature remained low. From the selected candidates, only montelukast shows a sufficiently high water-solubility to prepare a water-based solution. To achieve higher drug loads per ODF is ambitious, but is theoretically possible by printing multiple layers, using highly water-soluble APIs or highly loaded suspensions.

## 1. Introduction

Drug utilization research is an effective tool to identify the most used medications in patient groups, to assess the quality of prescribing and to facilitate rational drug use [1,2]. It is particularly useful in pediatrics, a heterogeneous group of patients who frequently use off-label medications [2]. In pediatrics, every age group has different needs and copes differently with the available administration forms [3]. For children, there is an apparent lack of age-appropriate dosage forms [2]. This results in manipulations of commercially available dosage forms, which may lead to medication errors [4]. Moreover, the adaptation of dosage forms may lead to invalidating the stability profile approved for the original product, altering taste and changing the biopharmaceutical behavior [5]. Furthermore, children aged 0 to 5 years have problems swallowing tablets and capsules.

The pharmaceutical development of medicines for pediatric use is stimulated by the European Medicine Agency [6], including the development of age-appropriate oral dosage forms, such as orodispersible films (ODFs). In short, ODFs are small polymer-based films that are placed in the oral cavity. They subsequently stick to the mucosa and disintegrate quickly. The active pharmaceutical ingredient (API) incorporated into the ODF is swallowed with the saliva and/or absorbed via the oral mucosa. ODFs cannot be spit out because of their sticky nature, and the risk of chocking is practically nil. In addition, ODFs can be prepared on a small scale (e.g., in a hospital or community pharmacy setting) [7], and are therefore considered patient-centric dosage forms [8] and have been accepted in pediatrics [9].

The most used preparation method for ODFs is the solvent casting technique [10]. With this technique, all excipients and APIs are mixed with or dissolved in a (co)solvent. The mixture is cast as a thin film onto a release liner, and is dried and cut into the desired size [7]. Besides this method, printing techniques are suitable for the incorporation of APIs onto ODFs, yielding a personalized oral dosage form [11,12]. Inkjet printing is an emerging technology in the pharmaceutical area, used for the direct labelling of dosage forms, but is also suitable for the API deposition [13]. The significant advantage of this technique is the flexible and precise deposition of the API on a substrate (such as a plain ODF) suitable for small-scale and continuous production [14]. In comparison with the casting of ODFs, less waste is produced. For printing, a pharmaceutical ink is used consisting of the API, a base liquid that easily evaporates and eventual further excipients. The dose is controlled by the drop volume, concentration of API in the ink, printed area, set resolution and number of layers. The prerequisite is that the target API is very well soluble in the base liquid (usually water) and remains stable during the printing process (stable at temperatures above room temperature).

Although medication use in children has been studied frequently, little attention had been paid so far to use these data for the identification of medications for age-appropriate and personalized administration forms such as ODFs. Therefore, in this study, the use of medication in pediatrics, children aged 0 to 5 years, in the Netherlands, is explored so as to identify suitable candidates for the development of ODFs as small-scale extemporaneous preparations. Next, the candidate APIs are examined regarding their suitability for inkjet printing.

## 2. Methods

### 2.1. Data Source

The data in this study are derived from the University of Groningen IADB.nl pharmacy dispensing database. It is a continuously expanding database that contains over 20 years of pharmacy dispensing data. These data are selected from approximately 60 community pharmacies and cover an estimated population of 600,000 patients in the Netherlands. The inclusion of pharmacy dispensing data in the database does not depend on the reimbursement status, or whether a specialist or general practitioner prescribes it. The dispensing data can be considered to be representative for the Netherlands, and has been widely used for drug utilization studies.

Each patient has a unique anonymous identifier; their date of birth and gender are known. In the Netherlands, generally, a patient is registered with one community pharmacy. Therefore, the medication records for each patient are virtually complete, except for over the counter (OTC) drugs and medication dispensed during hospitalization. Each person is individually tracked throughout the database period, and medication records contain information on the date of dispensing, the quantity dispensed, the dose regimen, the prescribing physician and the Anatomical Therapeutic Chemical classification code (ATC code) [15].

### 2.2. Selection of the Candidates for Development of ODFs

In this study, all of the dispensed medications for pediatrics (male and female), children from the age of 0 up until and including 5 years of age (on the dispensing date), were explored over a time period of five years (January 2013 until December 2017). All of the ATC codes dispensed were considered, except for B05 (blood substitutes and perfusion solutions), D (dermatological drugs), G01 (gynecological anti-infectives and antiseptics); J06 (immune sera and immunoglobulins), M02 (topical products for joint and muscular pain), P03 (ectoparasiticides, including scabidices, insecticides and repellents), S01 (ophthalmological drugs), V01AA (allergen extracts), V04 (diagnostic agents), V07 (all other non-therapeutic products), V08 (contrast media), V09 (diagnostic radiopharmaceuticals), V10 (therapeutic radiopharmaceuticals) and V20 (surgical dressings). Drugs or products under these ATC codes were considered as not being suitable as candidates for ODF formulation development.

After the selection of the pharmacy dispensing data, a stepwise approach with exclusion steps similar to the ones described in previous research was used to identify candidate APIs for ODF formulation development [1].

In the first step, medications that could not be identified because of an incomplete ATC code (e.g., only having a first level ATC code available in the database), had no legal status as drugs or were mentioned more than once in the pharmacy dispensing database (duplicates) were excluded. Furthermore, as the drug load per ODF is limited [13], all of the medications with a maximum drug load of more than 50 mg per dose were also excluded. The primary source for establishing the dose was the Dutch pediatric formulary [16]. If the dose was not mentioned in this formulary, the Dutch Informatorium Medicamentorum (IM) [17] or the Dutch Farmacotherapeutisch Kompas (FK) [18] were used.

In the second step, medications for non-oral administration routes were excluded. In the third step, off-label use medications were identified. These were, on the one hand, mentioned in the pediatric formulary but not intended for the age group, or, on the other hand, not mentioned in the pediatric formulary but mentioned in the IM or FK only for adult use. These can, however, be of interest for ODF formulation development and were included if dispensed regularly. In the fourth step, the availability of a commercial oral liquid or a standardized preparation in the Netherlands was investigated. These medications are not the first choice for ODF formulation development.

Finally, the manufacturing-related characteristics (water-solubility and taste) were retrieved from the literature [19,20] for all of the APIs that were considered potential candidates for ODF formulation. These characteristics were reviewed to evaluate their suitability for ODF formulation development via inkjet printing.

### 2.3. Ethical Statement

The study database IADB.nl uses de-identified medical records that could not lead to individual patients. According to the Code of Conduct for Health Research by the Foundation Federation of Dutch Medical Scientific Societies, approved by the Dutch Data Protection Authority in 2004 (Foundation Federation of Dutch Medical Scientific Societies 2004), no ethics committee approval is needed for research using anonymous medical records [21].

## 3. Results

The IADB.nl database included 53,542 patients in the age group of 0 to 5 years old. These patients received 227,898 dispensed medications over a period of fove years. This corresponded to 612 different APIs. The ratio of female:male patients was 52.2%:46.8%. Table 1 lists the medications per the ATC 1^st^ level.

In Table 2, the top 15 most dispensed medications are shown.

**Figure 1:**
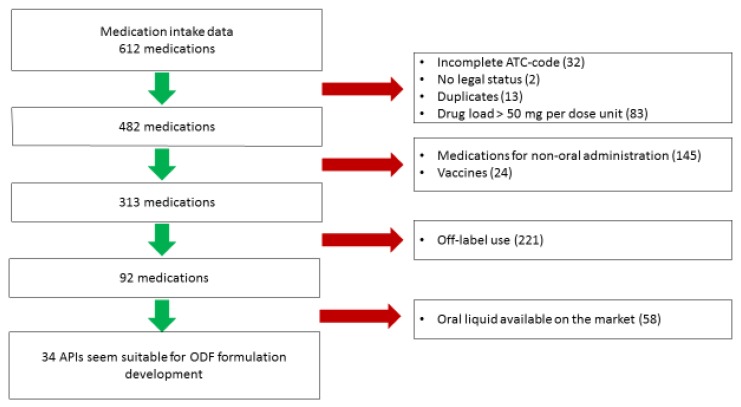
Flowchart for the selection of active pharmaceutical ingredients (APIs) towards orodispersible film (ODF) formulation development.

Medications were excluded based on the stepwise approach (see Figure 1). In the first step, 130 medications were excluded—32 medications had an incomplete ATC code, 2 medications had no legal status as drugs in the literature surveyed, 13 duplicates were removed and 83 medications contained a dose unit exceeding 50 mg.

In the second step, 169 medications were excluded. These were not intended for anoral route (e.g., for inhalation or nasal administration) and 24 medications were vaccines. In the third step, 221 medications were off-label dispensed and initially excluded.

For 92 medications, the availability of a commercial oral liquid or of a standardized preparation in the Netherlands was investigated. These medications were not the first choice for ODF formulation development. After these three selection steps, 34 APIs remained. In Table 3, an overview is given. The APIs were arranged according to water-solubility (g/L), from practically insoluble or insoluble to freely soluble (according to the definitions set by the European Pharmacopoeia 10^th^ edition) [19].

## 4. Discussion

The most frequently dispensed medications for pediatrics in the age group of 0–5 years were anti-infectives for systemic use. For almost 50% of the patients, the broad-spectrum antibiotic Amoxicillin was dispensed. This antibiotic is commonly used in the treatment of mild or moderate pneumonia or ear infections [16]. Furthermore, medications to treat disorders of the respiratory system (e.g., salbutamol) and medication to treat disorders of the alimentary tract were frequently dispensed (e.g., macrogol). The dose of antibiotics is usually too high to fit in an ODF.

Of the 612 medications, two had no legal status as medicine—biotin and ferrous chloride. Biotin is used as a food supplement for the treatment of biotinidase deficiency. Ferrous chloride is used as a trace element.

Vaccines were excluded in the second step. Vaccines are prone to degradation if taken orally and if exposed to high temperatures. They can, however, be stabilized with sugars, subsequently incorporated into ODFs and are absorbed via the buccal or sublingual route upon administration [13]. The printing of vaccines via inkjet printing is, because of the usually high temperatures involved, not possible. It could be feasible at low temperatures, if a bioprinter is used.

Of the 612 medications, 221 were not registered for pediatrics and thus used off-label. This is very common for the age group [2]. Off-label use is referred to by the European Commission as “any intentional use of an authorized product not covered by the terms of its marketing authorization and therefore not in accordance with the Summary of Product Characteristics”. This may, for example, be the use of a product for a different indication; the use of a different dosage, dosing frequency or duration of use; use of a different method of administration or use by a different patient group (e.g., children instead of adults) [22]. Off-label medications are prescribed if age-appropriate medications are not available. For health-care professionals, it is then the only option for treatment. Recently, the European Academy of Pediatrics and the European Society for Developmental Perinatal and Pediatric Pharmacology published recommendations on how and when to prescribe off-label medications [23].

Of the off-label dispensed medications, 220 were used by less than 10 patients, and for this reason, they were not the first choice for ODF formulation development. An example is gemfibrozil, which was used by one patient. It is indicated for the treatment of hypercholesterolemia in adults. The Summary of Products Characteristics of gemfibrozil states that use in children is not recommended because of a lack of data [24]. In the literature, however, use in pediatrics is described to treat neuronal ceroid lipofuscinosis [25,26]. One medication, ivermectin, was used by 38 patients. It was excluded for further research based on the safety aspect. In the literature, the safety of ivermectin in children weighing <15 kg has not been established [18]. Considering that the majority of children of 4–5 years of age have a bodyweight higher than 15 kg, ivermectin may be a candidate for further research if dispensed frequently.

After three exclusion steps, 92 medications were selected for further research. For 58 medications, a commercial oral liquid or an injection fluid (to be used as oral liquid) were available. Oral liquids have, for many years, been the dosage form of choice for pediatrics [27], and for that reason, these 58 medications were not regarded as the first choice for ODF formulation development. However, it is important to keep in mind that oral liquids have limitations, namely: formulations are not suitable for children with fluid restriction; the formulations possess a relatively short shelf life (due to chemical and/or microbiological stability limitations); accurate dosing is not always possible with the use of, e.g., a measuring cup and children may spit out the dose if the smell and taste is not acceptable for them [5,27,28]. Moreover, in case of a shortage of commercially available medications, age-appropriate dosage forms, such as ODFs, can be compounded and tailor-made in a pharmacy.

The acceptability of formulations for pediatrics have been under investigation since the development and establishment of the European Pediatric Regulation [6]. The taste is one of the key factors for the acceptance of a dosage form [29,30]. ODFs have been studied in children, and they positively respond to this dosage form [9]. The specific dimensions of the ODFs, the fast disintegration profile and the organoleptic properties contribute to their acceptability, and hence will positively influence medicine adherence [9].

The remaining 34 APIs were examined regarding their suitability for inkjet printing. A prerequisite is that the target API is very water soluble if water is used as base liquid, or otherwise, it is very soluble in the target base liquid. The more soluble the API, the higher the possible API content of the ink. Considering the list of final candidates for ODF formulation in pediatrics (Table 3), it is obvious that most of them are very slightly to practically insoluble in water. Only montelukast shows a sufficient water-solubility to prepare a water-based solution with a therapeutic effective strength. For the other candidates, a change in pH value, the addition of co-solvents and surfactants or the sole use of organic solvents may facilitate possibilities for formulation into a printed ODF.

The safety of the excipients in relation to the age group is an important issue in the development of a medicinal product [6]. Therefore, it must be ensured that the excipients are safe and suitable for use in pediatrics, and that the residual solvent content is below the accepted limit [14]. To circumvent limitations due to solubility, nanosuspension inks can be prepared [31,32]. The most critical issue with inkjet printing is the clogging of nozzles by particles of an inappropriate size, and the recrystallized API or excipients due to the solvent evaporating too quickly. Failed nozzles lead to an underdosing of the API. Moreover, it must also be ensured that the ODFs are not damaged or dissolved by the ink applied. There is a possibility to create porous ODFs for higher liquid absorption, containing mesoporous silica particles or foamed hydroxypropyl methylcellulose [33,34,35]. However, the in vivo profile has to be investigated if the mouthfeel stays acceptable and if the dissolution profiles change.

Compared with adults, the oral cavity in pediatrics is small. Therefore, small ODF sizes (e.g., 1.8 × 1.8 cm) are preferred. Consequently, the printed ODF area and thus drug load is limited. It should however be feasible to incorporate 25 mg into a small size ODF, by printing multiple layers and using highly water soluble APIs or highly loaded suspensions. Higher dosages can only be achieved by the intake of several ODFs at the same time. A dosage of, e.g., 50 mg implies the intake of two ODFs, which should be acceptable.

From the final selection of APIs, diclofenac [36], indomethacin [32,37], montelukast [38] and prednisolone (frequently dispensed instead of prednisone, as listed in Table 3) [39] have been already successfully printed using the inkjet technique on ODFs or transparency sheets. In the case of montelukast and prednisolone, the therapeutic dose was achieved, however, in the case of diclofenac and indomethacin, the printed dose remained too low. An option is, as mentioned before, to print multiple layers; however, this may lead to morphological changes of the film substrates [37]. Pharmaceutical inkjet printing is a suitable technique for ODF manufacturing for pediatric application. The literature reveals that it is possible to print therapeutically applied doses of a number of the selected APIs [38,39].

## 5. Conclusions

In conclusion, the most dispensed medications in the target group were antibiotics and off-label use medications, which is very common in pediatrics.

From the database, 34 APIs were identified as candidates for ODF formulation development via inkjet printing. Only montelukast showed sufficient water-solubility to prepare a water-based solution of a therapeutically effective strength. For most of the APIs, organic solvents are needed or nanosuspensions can be used. To achieve higher drug loads per ODF is ambitious, but is theoretically possible by printing multiple layers and using highly water soluble APIs or highly loaded suspensions.

Establishing inkjet printing technology in pharmacies requires a good process understanding as well as guaranteed safety with regard to the residual solvents in the final product. The printing of ODFs is, however, a step forward in the development of age-appropriate and thus individualized dosage forms.

## Figures and Tables

**Table 1:** Distribution of the dispensed medications per the Anatomical Therapeutic Chemical classification code (ATC code) 1^st^ level (ordered according to the frequency of dispensed medications).

ATC Level	Number of Dispensed Medications (% of Total Dispensed Medications)
J (anti-infectives for systemic use)	67,292 (29.5%)
R (respiratory system)	59,984 (26.3%)
A (alimentary tract and metabolism)	58,327 (25.9%)
S (sensory organs)	13,338 (5.9%)
N (nervous system)	11,619 (5.1%)
H (systemic hormonal preparations, excluding sex hormones and insulins)	4202 (1.8%)
C (cardiovascular system)	4109 (1.8%)
B (blood and blood forming organs)	3184 (1.4%)
M (musculoskeletal system)	1967 (0.9%)
P (antiparasitic products, insecticides and repellents)	1288 (0.6%)
L (antineoplastic and immunomodulating agents)	1286 (0.6%)
G (genitourinary system and sex hormones)	1180 (0.5%)
V (various)	122 (0.05%)

**Table 2:** The 15 most dispensed medications, with their ATC codes, number of users and percentage of users (calculated on the total amount of patients, in the age group of 0 until and including 5 years).

	ATC Code	Medications	Number of Users (n)	Percentage of Users (%)
1	J01CA04	Amoxicillin	25265	49.2
2	R03AC02	Salbutamol	11150	20.8
3	A07AA02	Nystatin	6767	12.6
4	A06AD15	Macrogol	5338	10
5	J01CR02	Amoxicilline plus β-lactamase inhibitor	4514	8.4
6	S01AA01	Chloramphenicol	3895	7.3
7	R06AX27	Desloratidine	3685	6.9
8	J01FA10	Azithromycine	3376	6.3
9	A06AD11	Lactulose	3272	6.1
10	S01AA13	Fusidic acid	3244	6.1
11	R01AA07	Xylometazoline	2949	5.5
12	R03BA05	Fluticasone	2739	5.1
13	A06AD65	Macrogol, combinations	1687	3.2
14	J01CF05	Flucloxacillin	1679	3.1
15	J01FA09	Clarithromycin	1576	2.9

**Table 3:** Candidates for ODF formulation development via inkjet printing, arranged by water-solubility.

	ATC Code	Name	Water Solubility (g/L)	Taste	Number of Users (n)	Daily Dose **
1 *	N06AA04	Clomipramine	0.000294	n/a	1	0.25–0.5 mg/kg/day in two doses
2	M01AB01	Indomethacin	0.000937	Slightly bitter	4	2 mg/kg/day in two to four doses
3	A06AB02	Bisacodyl	0.00127	Tasteless	340	5 mg/day
4	L04AA18	Everolimus	0.00163	n/a	2	1.6–9 mg/m^2^/day
5	M01AB05	Diclofenac	0.00237	n/a	754	1–3 mg/kg/day, in two to four doses
6	C08DA01	Verapamil	0.00447	n/a	3	3–8 mg/kg/day, in three doses
7	A11CC04	Calcitriol	0.00667	n/a	1	0.01–0.1 mics/kg/day, in two doses
8	N02CX01	Pizotifen	0.00706	n/a	1	0.5–1.5 mg/day, in one to three doses
9	N05CD08	Midazolam	0.00987	Bitter	157	0.2–0.5 mg/kg
10	N05AG02	Pimozide	0.01	n/a	1	0.5–1 mg/day
12	N06AA02	Imipramine	0.0182	n/a	3	20–30 mg/day
13	N04AA02	Biperiden	0.0251	n/a	9	1–2 mg, one to three times daily
14	N05CD02	Nitrazepam	0.0299	n/a	9	2.5–5 mg/day
15	N05BA01	Diazepam	0.050	Bitter aftertaste	501	0.1–0.8 mg/kg/day, in four doses
y16	H02AB07	Prednisone	0.07754	Bitter aftertaste	8	1–2 mg/kg, in two doses
17	C05AA12	Triamcinolone	0.080	Bitter	2	<35 kg: 4–12 mg/day
18	H01BA02	Desmopressin	0.11	n/a	43	0.2 mg/day
19	C03BA04	Chlorthalidone	0.12	n/a	3	0.5–1 mg/kg, max 1.7 mg/kg per 48 h
20	H02AA02	Fludrocortisone	0.14	n/a	11	50–150 mics/day, in two doses
21	N03AX09	Lamotrigine	0.17	n/a	35	0.3–1.2 mg/kg
22	N05BA04	Oxazepam	0.179	Bitter	27	10–30 mg per day, in three to four doses
23	A02BC01	Omeprazole	0.359	n/a	453	1 mg/kg/day in one to two doses, max 40 mg/day
24	J04BA02	Dapsone	0.38	Slightly bitter	2	1–1.5 mg/kg/day
25	N05BB01	Hydroxyzine	0.428	Bitter	7	1–2 mg/kg/day, max 100 mg/day
26	N07CA02	Cinnarizine	0.75	Bitter	3	12.5 mg, if necessary, three to four times per day
27	R06AE05	Meclozine	1	tasteless	1	6.25 mg, if necessary, three to four times per day
28	N07AA02	Pyridostigmine	1.04	tasteless	18	10 mg/day, max 100–500 mg in three doses
29	N02AE01	Buprenorphine	1.680	Bitter	3	15 mics/kg/day in three doses
30	L04AX03	Methotrexate	2.6	n/a	28	10–15 mg/m^2^/dose, once per week
31	V03AH01	Diazoxide	2.85	n/a	1	5 mg/kg/day, in two doses
32	N03AX11	Topiramate	9.8	Bitter	11	1–3 mg/kg/day
33	M04AC01	Colchicine	10	n/a	7	0.5–1 mg/day, in one to two doses
34	R03DC03	Montelukast	>100	n/a	178	4 mg per day

* <0.1 g/L = practically insoluble or insoluble (red); 0.1–1 g/L = very slightly soluble (orange); 1–10 g/L = slightly soluble (dark yellow); 30–100 g/L = sparingly soluble (light yellow, not applicable in this table); 100–1000 g/L = freely soluble (green). ** [16,17,18].
